# Hypertension prevents a sensory stimulation-based collateral therapeutic from protecting the cortex from impending ischemic stroke damage in a spontaneously hypersensitive rat model

**DOI:** 10.1371/journal.pone.0206291

**Published:** 2018-10-23

**Authors:** Aneeka M. Hancock, Ron D. Frostig

**Affiliations:** 1 Department of Neurobiology and Behavior, University of California Irvine, Irvine, California, United States of America; 2 Center for the Neurobiology of Learning and Memory, University of California Irvine, Irvine, California, United States of America; 3 Department of Biomedical Engineering, University of California Irvine, Irvine, California, United States of America; Fraunhofer Research Institution of Marine Biotechnology, GERMANY

## Abstract

Assessing potential stroke treatments in the presence of risk factors can improve screening of treatments prior to clinical trials and is important in testing the efficacy of treatments in different patient populations. Here, we test our noninvasive, nonpharmacological sensory stimulation treatment in the presence of the main risk factor for ischemic stroke, hypertension. Utilizing functional imaging, blood flow imaging, and histology, we assessed spontaneously hypertensive rats (SHRs) pre- and post-permanent middle cerebral artery occlusion (pMCAO). Experimental groups included a treatment SHR group (sensory-stimulated group), control untreated SHR group (no sensory stimulation), and a treated (sensory-stimulated) Wistar-Kyoto normotensive group. Unlike our previous studies, which showed sensory-based complete protection from impending ischemic cortical stroke damage in rats as seen in the treated Wistar-Kyoto group, we found that SHRs at 24hr post-pMCAO lacked evoked cortical activation, had a significant reduction in blood flow within the MCA, and sustained very large infarcts regardless of whether they received stimulation treatment. If translatable, this work highlights a potential need for a combined treatment plan when delivering sensory stimulation treatment in this patient population.

## Introduction

The heterogeneity of stroke has made the discovery of viable therapeutics challenging. Despite the fact that most stroke patients present with at least one risk factor, many studies do not take into account comorbidities when developing new treatments. Thus, it’s important to address a crucial discrepancy between bench and bedside and test potential therapeutics in the presence of these risk factors [1,2], as this is often a reason that clinical trials fail for otherwise promising treatments [3].

With approximately 77% of stroke patients having hypertension, it is the number one risk factor for ischemic stroke [4,5]. Prolonged periods of hypertension can lead to changes in the cerebral vasculature, but despite extended periods of high blood pressure causing pulsatile stress on the arterial trees, the major complication of hypertension is thrombotic, rather than hemorrhagic [6]. This thrombotic paradox of hypertension could be due to hypercoaguability or a prothrombotic state due to an imbalance between coagulation and fibrinolytic pathways, and high pressures damaging vessel walls [6,7]. Hypertension can also lead to stroke by promoting atherosclerosis, arteriosclerosis, and lipohyalinosis [8,9], resulting in maintenance of the elevated blood pressure [10], and thrombotic or embolic stroke [9,11]. Additional adaptive structural changes can occur due to increased workload, such as growth of smooth muscle cells that can reduce vessel lumen diameter [11,12]. This can further compound the vascular impairments as it results in increased vascular resistance that can reduce collateral blood flow, resulting in ischemia distal to an arterial occlusion [13], and reduction of salvageable penumbra.

It is clear that hypertension, as it relates to stroke pathology, is a complex issue, and greatly contributes to the heterogeneity of the stroke population. The development of new stroke therapeutics is imperative given that there is no cure, and various therapeutic targets could provide protection from stroke depending on patients’ comorbidities. Currently, the only FDA-approved treatment, tPA, results in positive outcome in only a small portion of stroke patients due to its limited therapeutic window and the risk of hemorrhagic transformation. Recent advances have enabled improved tPA efficacy by applying tPA with concurrent recanalization via endovascular thrombectomy, resulting in increased responsiveness to treatment but implementation requires extensive expertise [14]. Our lab has shown that a collateral-based sensory stimulation treatment is a promising therapeutic treatment, as it is noninvasive, has the potential to be delivered immediately, has long-lasting effects, and completely prevents impending ischemic stroke damage [15–19]. Additionally, we have already tested whether rats can be protected in the face of one risk factor, old age, and have observed complete protection in rodents that are equivalent in age to typical stroke patients (65 years old) [20]. However, to further assess the translational potential of this collateral-based treatment and potential limitations, it’s important to test it in cases where vessel lumen diameter, and other vascular characteristics, is negatively altered, as is the case in hypertension. Thus, in this study we aimed to test whether spontaneously hypertensive rats (SHRs), a widely used model of essential hypertension, could be protected from ischemic damage following an identical stimulation protocol that protects normotensive rats.

## Materials and methods

All procedures were in compliance with NIH guidelines and approved by UC Irvine Animal Care and Use Committee (protocol #: 1997–1608, assurance ID#: A3416.01), and in compliance with the ARRIVE guidelines.

### Subjects and surgical preparation

Fourteen experimental subjects, 295-400g (4–5 months of age) male spontaneously hypertensive rats (Harlan Laboratories, Indianapolis, IN, USA) with systolic blood pressures of ~150 mmHg, were individually housed in standard cages. At the beginning of each experiment, subjects were injected intraperitoneally with a Nembutal bolus (55 mg/kg b.w.). Supplemental injections of Nembutal (27.5 mg/kg b.w.) were given as necessary. After resection of soft tissue, a ~6.5 x 8 mm ‘imaging’ area of the skull over the left primary somatosensory cortex (rostromedial corner positioned approximately 1mm caudal and 2mm lateral from bregma) was thinned to ~150μm using a dental drill. 5% dextrose (3mL) and atropine (0.05 mg/kg, b.w.) were administered at the beginning of the experiment and every six hours after until the animal was returned to its home cage. Body temperature was measured via a rectal probe, and maintained at 37° Celsius by a self-regulating thermal blanket. Animals were returned to their home cage and allowed to recover overnight prior to all +24 hour experimentation.

### Overview

Using a within subject design that is identical to our previous studies, 14 subjects were randomly assigned to a +0h group (i.e., immediate sensory stimulation period following pMCAo) or a no-stimulation control group. Baseline functional imaging was collected for all subjects at the beginning of surgery. All +0h subjects (n = 7) then received a pMCAO, and immediate post-occlusion whisker stimulation. Post-occlusion whisker stimulation consisted of 1 s of 5 Hz deflections of a single whisker (whisker C2). This stimulation was intermittently delivered (with random intervals averaging 21 seconds) 256 times, totaling 4.27 minutes of stimulation, over the course of 2 hours [15]. No-stimulation controls (n = 7) underwent identical pMCAO to that of +0h subjects, but never received whisker stimulation; pMCAO was immediately followed by a 5-hour no-stimulation period. The length of this 5 hour quiet period was chosen by Lay et al. [15] to match time under anesthesia to subjects in that study that received the two hours of whisker stimulation three hours post-occlusion–this has become the standard for our no-stimulation controls as it produces invariable cortical infarct in these subjects. After whisker stimulation or quiet period, all rats were placed back in their home cage for recovery, until their follow-up assessment at 24 hours post-pMCAO, which consisted of functional imaging and blood flow imaging. At the end of pMCAO surgery, rats received subcutaneous ampicillin antibiotic injections (100 mg/kg), and Flunixin meglumine analgesic was injected subcutaneously (2 mg/kg). The closed wound was covered with topical antibiotic, and rats were monitored while recovering from anesthesia. At the end of 24 hour imaging, rats were then euthanized and the brains were removed for histological assessment. No mortality has been encountered in any of the experimental groups.

### Permanent middle cerebral artery occlusion

Permanent ischemic conditions are achieved as follows: The base of the left proximal middle cerebral artery at the M1 segment is permanently occluded [21–24] blocking flow to all MCA cortical branches. To do this, the skull and dura are carefully removed from a 2x2mm ‘surgical window’ just anterior and lateral to the imaging window (over the M1 segment of MCA, just distal to MCA’s lenticulostriate branches and proximal to any cortical branching). A half-curve reverse cutting suture needle is cut in half and threaded with two 4–0 silk threads and passed through the pial layer of the meninges, below MCA (the needle is kept above the cortical surface to the extent possible to prevent damage). Then the two threads (moved to ~1mm apart after being strung beneath the artery) are both tied and tightened around MCA and the vessel is transected (completely severed) between the two knots. Care is taken to avoid damaging the artery, and experiments are terminated if there are signs of bleeding from MCA. This method of occlusion has been reviewed by Davis et al. [25].

### Histology (2,3,5-triphenyltetrazolium chloride staining for infarct)

At the conclusion of each experiment, rats were euthanized with sodium pentobarbital (2–3 mL, intraperitoneally), the brain was removed, sectioned into 2 mm coronal slices, and incubated in 2% 2,3,5-triphenyltetrazolium chloride at 37°C for 20 min in the dark [26]. TTC is enzymatically reduced, producing formazan (a bright red byproduct), by dehydrogenases in active mitochondria. Red stain intensity correlates with the number and functional activity of mitochondria, unstained (white) areas are indicative of infarct [27]. The TTC-stained sections are photographed with a digital camera, and images are analyzed using ImageJ software. The total infarct volume is determined by multiplying the infarct area of each slice by the thickness of that slice. Final volumes are then corrected for edema. An observer blind to experimental condition performs this volume calculation. A small surgical lesion (<1 mm in diameter) is occasionally apparent at the immediate site of MCA occlusion. This occurs infrequently and equivalently in all experimental groups (1–2 subjects per group). The small amount of damage occasionally produced at the surgical site can be readily distinguished from the large ischemic infarct and is excluded from infarct analysis [22].

### Intrinsic signal optical imaging (ISOI) and analysis

A detailed description of ISOI data acquisition and analysis can be found elsewhere [28,29]. Briefly, a charge coupled device (CCD) camera (either a 16-bit Cascade 512F or a 12-bit Quantix 0206, Photometrics, Tucson, AZ, USA) equipped with an inverted 50 mm AF Nikon lens (1:1:8, Melville, NY, USA) combined with an extender (model PK-13, Nikon, Melville, NY, USA) is used for imaging and controlled by V++ Precision Digital Imaging System software (Digital Optics, Auckland, NZ). During each 15-s trial, 1.5 s of prestimulus data followed by 13.5 s of poststimulus data is collected, with a 6±5 sec random inter-trial interval. Stimulus consists of a single whisker being deflected by 9° in the rostral-caudal direction at a rate of 5 Hz for a total stimulus duration of 1 second. The cortex is illuminated with a red light emitting diode (635 nm maximum wavelength). Data are collected in blocks of 64 stimulation trials, and a sampled time point (for example pre-pMCAO baseline) is considered complete upon summation of 128 stimulation trials. Ratio images are created from calculating fractional change (FC) values by dividing each 500ms frame of poststimulus signal activity by the 500ms frame of prestimulus intrinsic signal activity collected immediately before stimulus onset. The ratio image containing the maximum areal extent for the first intrinsic signal phase (Initial Dip) is Gaussian filtered (half width = 5) and the areal extent quantified at a threshold level of 2.5 x 10^−4^ away from zero. Peak amplitude is quantified in fractional change units from the pixel with the peak activity within the maximum areal extent for both of the intrinsic signal phases.

### Laser speckle imaging (LSI) and analysis

A detailed description of LSI [30,31] data acquisition and analysis can be found elsewhere [15]. Briefly, a 632.8 nm 15 mW HeNe laser was used as the illumination source. The speckle pattern from the 5.12×5.12 mm imaged region was captured as 512×512 pixel images by a 16-bit CCD camera (Cascade 512F) equipped with a Navitar zoom lens plus extenders such that speckle size matched camera pixel size. Collected images were processed as previously described [15]. Speckle contrast images were converted to speckle index images by calculating their inverse squares multiplied by the exposure time in seconds, so that larger index values corresponded to faster blood flow. Speckle index images were then averaged to improve signal-to-noise ratio. To quantify blood flow within the MCA, we calculated the mean value within a region of interest (ROI) in MCA cortical branches as defined according to several criteria described previously [15]. All flow index values were scaled over a range where 0 flow was set at noise values. Values were collected from all euthanized animals 5 minutes after the cessation of the heart beat, and these noise values were subtracted from all other values.

### Statistical analysis

For imaging data, analysis of variance (ANOVA) were run on baseline values to ensure no significant differences before pMCAO. Because there were no responses to quantify at 24 hours, post-pMCAO imaging evoked area and amplitude were converted to difference score values (postocclusion—baseline) with values away from 0 signifying a change from baseline. A constant was added to difference values, which were then transformed with a natural log function to better satisfy the assumptions of ANOVA and inferential statistics were performed on the transformed data. Raw values of laser speckle velocity were used for analysis. After ANOVA, specific contrasts were performed to identify which groups differed from baseline. Alpha level was set to 0.05 and Bonferroni adjustments were applied to account for multiple contrasts. Infarct volume comparisons were performed by employing two-sample t-tests. All plotting and statistics were performed using SYSTAT 11 (SYSTAT Software Inc., Chicago, IL, USA).

## Results

### Treated hypertensive rats do not exhibit protection of cortical function

**Fig 1:**
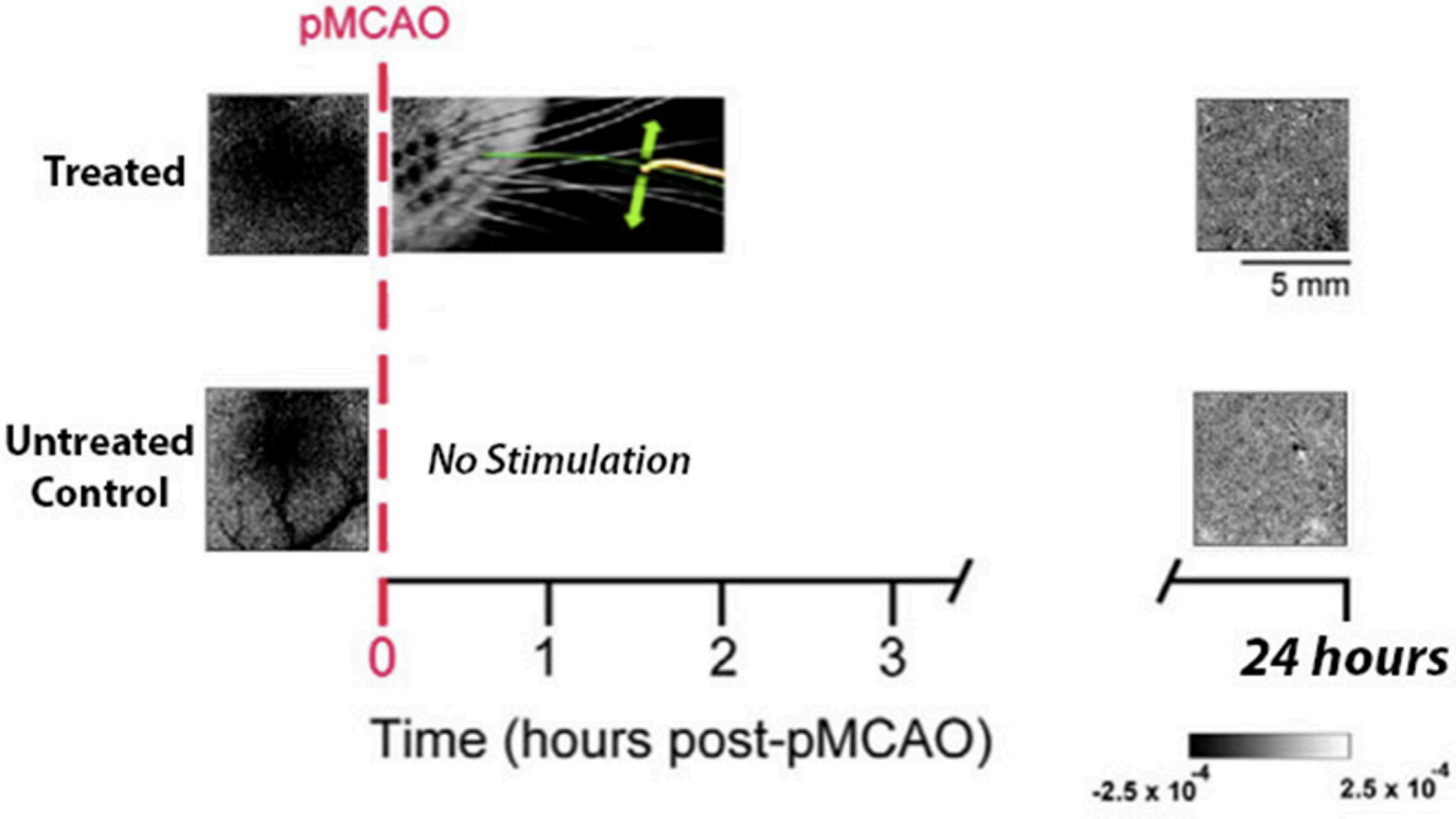
Treated hypertensive rats lack cortical activity at 24 hours post-pMCAO. Experimental schema with ISOI representative cases for treated (top) and untreated (bottom) subjects, before (left) and 24 hours after (right) pMCAO. Treated (n = 7) and untreated (n = 7) subjects have normal whisker functional representations (WFR) at baseline. After imaging, all subjects received a pMCAO (dashed vertical red line), which was followed immediately by 2 hours of whisker stimulation treatment or a no-stimulation period. At 24 hours post-pMCAO, all subjects lacked a WFR. Green arrows indicate the direction of whisker movement during stimulation.

Before pMCAO, there was no significant difference between groups for either the area (mean_treated_ = 5.01 ± 1.11; mean_untreated_ = 4.18 ± 0.80; F_1,12_ = 0.19, P > 0.05, ANOVA) or amplitude (mean_treated_ = 4.43±0.28; mean_untreated_ = 4.13 ± 0.26; F_1,12_ = 0.55, P > 0.05, ANOVA) of the whisker functional representation (WFR). At twenty-four hours post-pMCAO, neither treated or untreated groups were protected and therefore had no whisker functional representation at this time point (Fig 1).

**Fig 2:**
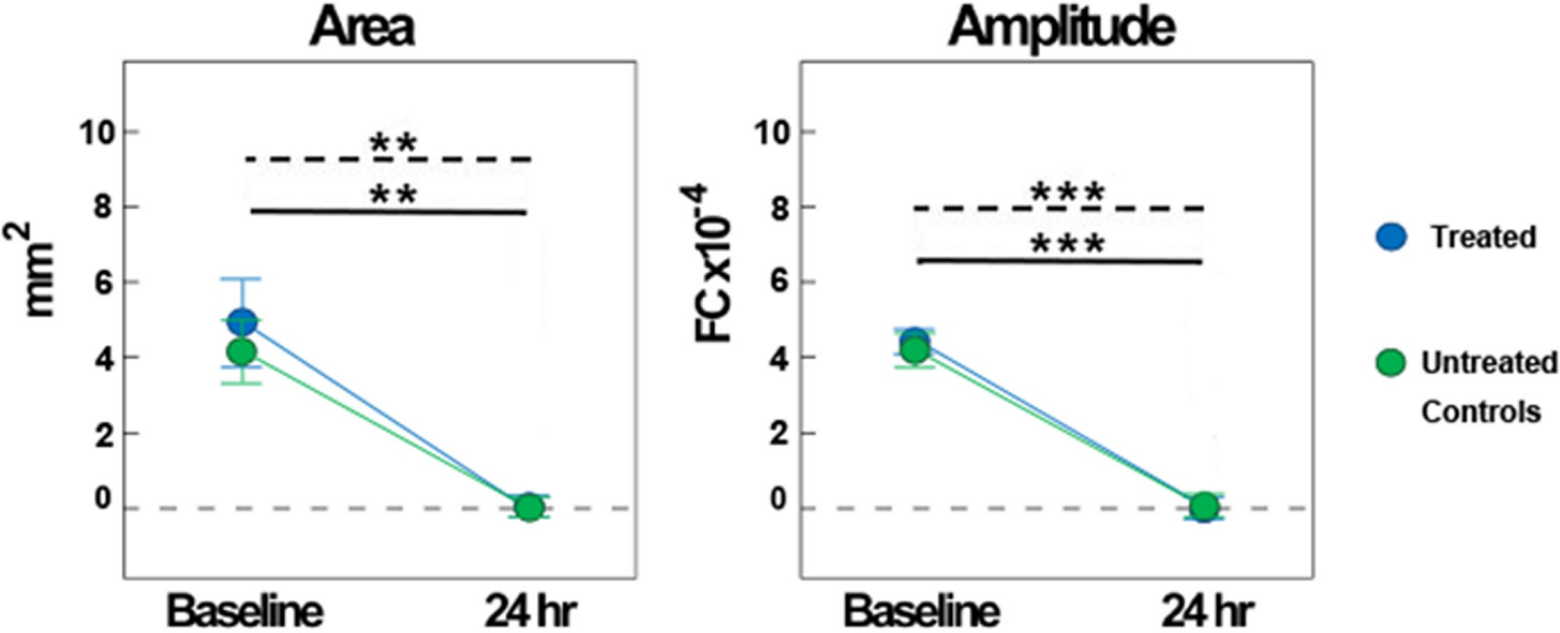
Treated and untreated hypertensive subjects show a strong reduction in the whisker functional representation (WFR) 24 hours after pMCAO. Quantification of the area (left) and amplitude (right) of the WFR at both baseline and 24 hours for treated and untreated subjects. Treated (n = 7) and untreated (n = 7) subjects showed a significant decrease in both parameters at 24 hours post-pMCAO compared to baseline (dashed and solid lines, respectively) (* = p ≤ 0.05, ** = p ≤ 0.01, *** = p ≤ 0.001).

As such, there were no significant differences between groups at 24 hours for either area (mean_treated_ = 0.02 ± 0.02; mean_untreated_ = 0.01 ± 0.01; F_1,12_ = 0.79, P > 0.05, ANOVA) or amplitude (mean_treated_ = 0.24±0.17; mean_untreated_ = 0.24±0.16; F_1,12_ = 0.37, P > 0.05, ANOVA). The lack of cortical activity at twenty-four hours post-pMCAO resulted in a significant reduction in area and amplitude compared to baseline activity for treated subjects (n = 7; area: F_1,12_ = 13.97, P < 0.005; amplitude: F_1,12_ = 83.02, P < 0.001) as well as untreated subjects (n = 7; area: F_1,12_ = 10.14, P < 0.01; amplitude: F_1,12_ = 68.08, P < 0.001) (Fig 2). Thus, treated and untreated subjects were equivalent, as treatment did not have a protective effect.

### Hypertensive rats have strongly reduced retrograde collateral flow feeding the MCA at 24 hours post-pMCAO

**Fig 3:**
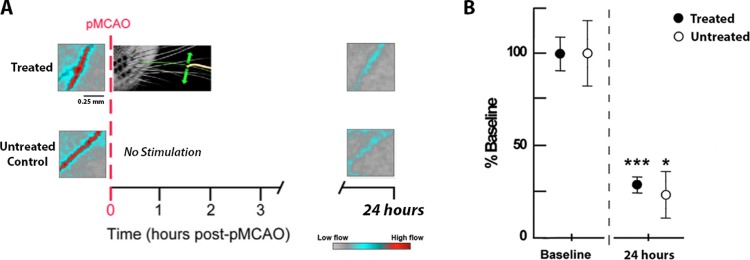
Treatment resulted in minimal retrograde blood flow within the MCA at 24 hours post-pMCAO. A, Experimental schema with LSI representative cases for treated (top) and untreated (bottom) subjects, before (left) and 24 hours after (right) pMCAO. Treated (n = 4) and untreated (n = 3) subjects are similar in that there was reduced blood flow at 24 hours post-pMCAO. B, Quantification of laser speckle velocity for both groups, represented as a percentage of baseline values. The reduction in blood flow was significant for all subjects. Green arrows indicate the direction of whisker movement during stimulation.

A subset of subjects underwent blood flow imaging to assess whether the MCA was capable of being reperfused via the collateral vasculature. There were no significant differences between groups either before (F_1,5_ = 3.83, P > 0.05, ANOVA) or twenty-four hours (F_1,5_ = 0.05, P > 0.05, ANOVA) after pMCAO. We found that treated (n = 4; mean_%ofbaseline_ = 31.62) and untreated (n = 3; mean_%ofbaseline_ = 24.33) subjects all had reduced blood flow within the MCA post-occlusion, and this was a significant reduction compared to baseline flow for both treated (t(3) = [18.63], p = [0.0003]) and untreated subjects (t(2) = [4.612], p = [0.0439]) (Fig 3).

### Hypertensive rats sustain large cortical infarcts after pMCAO despite receiving immediate treatment

**Fig 4:**
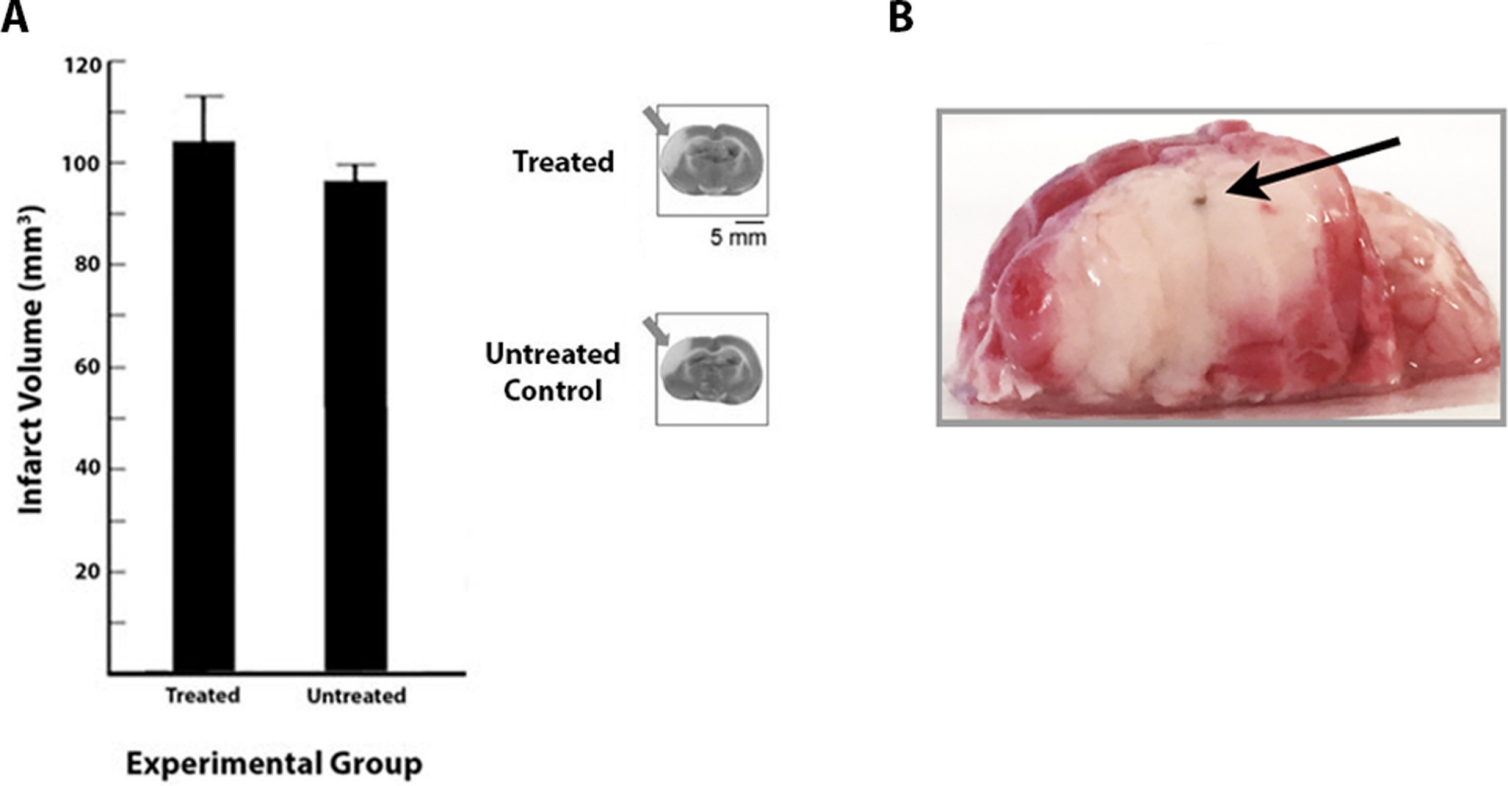
Hypertensive rats are not protected from impending ischemic damage and sustain large infarcts. A, Quantification of infarct and representative TTC-stained sections for treated and untreated subjects. No difference in infarct size existed between the groups (n = 7 per group). Gray arrows indicate cortical infarct. B, A representative example of TTC staining in the whole brain following the addition of all individually stained 2mm sections. This illustrates that the entire MCA territory was infarcted. Ink spot (black arrow) indicates the location of the center of the barrel cortex.

Finally, we assessed ischemic damage with TTC staining and found that the hypertensive rats sustained large infarcts regardless of whether they received immediate treatment (Fig 4).

### Wistar Kyoto rats

Wistar Kyoto (WKY) rats are commonly used as controls for SHR. We have therefore run +0h experiments on a group (n = 6) of WKY rats. Their TTC results showed no sign of infarct in their cortex (see S1 Fig for a TTC example), consistent with our previous +0h findings in Sprague Dawley rats. Two WKY rats showed some subcortical damage, damage not typically seen in +0h SHR. More research is needed to reveal whether WKY rats are potentially more susceptible for subcortical infarct under these experimental conditions.

There was no effect of treatment on infarct size (t[12] = 0.76, *P*>0.05), with treated subjects sustaining infarcts ranging from 74.27–137.94 mm^3^ (mean = 104.05±9.37 mm^3^), and infarcts for untreated subjects ranging from 83.96–107.74 mm^3^ (mean = 96.53±3.32 mm^3^).

## Discussion

Our previous research has shown that in normotensive rats, cortical activity is maintained in early-treated, protected subjects at 24 hours post-pMCAO [15,16,18]. This finding stands in stark contrast to the results presented here. It is clear from the ISOI, LSI and TTC data that these hypertensive rats are not protected from impending ischemic stroke damage when given immediate collateral-based sensory stimulation treatment. The whisker functional representation for treated subjects was completely eliminated at 24 hours post-pMCAO. Importantly, this is identical to what we observed for untreated control subjects that never received treatment. Additionally, these treated subjects lacked significant retrograde blood flow within the MCA at 24 hours post-pMCAO as we normally observe in normotensive treated, protected subjects [16]. Finally, TTC staining confirmed that these subjects did in fact have infarcts that seemed to span the entire MCA territory. Not only did treated subjects sustain massive infarcts similar to the untreated controls, but these values are much larger than what we have observed in any of our previous normotensive subjects when anesthetized with pentobarbital. Normotensive untreated controls typically sustain infarcts of 28.4±2.4 mm^3^, while subjects that receive treatment 3 hours post-pMCAO (+3h) sustain larger infarcts of 61.4±2.4 mm^3^ [15], which is still small compared to the infarct volumes reported here. The only damage that treated subjects tend to experience comes from surgical damage (<1mm in diameter) and is observed infrequently.

Hypertension is the single most important risk factor for the development of stroke [32], as it promotes atherosclerosis and various types of structural remodeling as compensatory mechanisms for dealing with chronic high blood pressure. Unfortunately, all of these vascular stresses can result in stenosis and increase the risk of ischemic stroke. To further compound the issue, hypertension can alter cerebrovascular autoregulation and reduce vascular responsiveness to endothelium-dependent vasodilators such as nitric oxide [33,34] and functional hyperemia [12,35–38]. In some cases, this impaired functional hyperemia is due to stenosis, and thickening and hardening of vessel walls, but it could also be attributed to high angiotensin II levels [39]. These effects are present both in animal models and in patients with hypertension. A study by Jennings et al. [35] showed that increased cerebral blood flow as a result of functional hyperemia from brain activation was reduced in patients with chronic hypertension. Additionally, several studies in humans have also shown that a history of hypertension is more frequently associated with fewer leptomeningeal collaterals in CT angiography [40], and with poor collateral flow [41].

Given that hypertension is known to negatively impact the brains vasculature in humans and animal models of hypertension, including the collateral vessels that are critical to the efficacy of our treatment, it is not surprising that these hypertensive rats were not protected from impending ischemic stroke damage. The model used here, the spontaneously hypertensive rat (SHRs), was first developed in 1963 by Okamoto and Aoki as a model for essential hypertension, which is the most common form (i.e., persistent high blood pressure of unknown causation), affecting approximately 95% of hypertensive patients. SHRs are currently the only, and thus most widely used, model for human essential hypertension [42], since it occurs without any treatment to induce hypertension [43]. SHRs also exhibit hypertension in stages similar to humans, with pre-hypertension existing for the first 6–8 weeks of their lives (with systolic blood pressures around 100–120 mmHg), followed by hypertension developing over the next 12–14 weeks, where systolic blood pressure remains over 150 mmHg [42]. The cerebral vasculature of these rats is known to have similar characteristics to that of hypertensive individuals. This includes decreased distensibility of cerebral arteries due to an increase in collagen but not elastin content [44], and increased thickness of the vascular wall, specifically the intima media (which comprises the middle smooth muscle, and inner endothelial layers of a blood vessel) [32,45,46]. This is in contrast to large amounts of smooth muscle, and small amounts of elastin, basement membrane and collagen in pial arteries in normotensive rats.

Due to these hypertension-induced vascular impairments, spontaneously hypertensive rats sustain infarcts of greater volume, but with less variability, than normotensive controls [47,48]. This is likely the result of cortical collateral flow that’s been reduced to an unsustainable level due to the reduced luminal diameter and reduced vasodilation and autoregulation capabilities rather than a lack of collaterals [12,49–51]. The severely impaired vasculature in these rats could thus explain the larger infarct size compared to normotensive untreated and +3h subjects (those that receive sensory stimulation treatment 3 hours post-pMCAO) from our lab. +3h subjects may have large infarcts due to enhanced blood flow through functioning collaterals at a late time point, leading to reperfusion injury and thereby exacerbating the damage that has already occurred, while the hypertensive rats may have a general reduction in blood flow post-occlusion due to increased vascular resistance as a result of brain-wide vascular impairments and reduced vessel lumen diameters. This could result in a larger portion of cortex remaining hypoperfused after pMCAO, and ultimately evolution of the penumbra into infarct.

The findings presented here further corroborate the existing literature. Regardless of whether rats received early sensory stimulation treatment, we observed severely reduced collateral flow in hypertensive rats as demonstrated by significantly reduced blood flow in the MCA at 24 hours post-pMCAO. This reduction in retrograde blood flow in the MCA, which we have shown originates from the pial collaterals in treated, protected subjects [15], resulted in the development of ischemic damage as is evident by the lack of evoked cortical activity and large infarcts.

The collateral vasculature is known to be responsible for rescuing penumbral tissue and reducing infarct size. However, our results show that cerebrovascular impairments in these hypertensive rats did not permit viable levels of collateral blood flow and led to ischemic damage, at least when using the same protective whisker stimulation parameters employed in normotensive rats, which may not be sufficient for protection in SHRs. When considering the translational potential of this collateral-based treatment for ischemic stroke patients, it is important to keep in mind that these rats may not be an optimal model, as they begin developing hypertension from a relatively young age. Thus, although it appears that this treatment might not be a viable solution to prevent ischemic damage in this population of stroke patients, it is possible that the effects of hypertension in these rats may impair the cerebral vasculature more severely compared to patients with hypertension. Additionally, this model does not take into account the fact that many patients are on medications to control blood pressure, which may also reduce the severity of the vascular impairments depending on how long the patient has had hypertension and the length of time that they’ve been taking medications. Thus, an effective solution for patients with hypertension and minimal collateral flow may be to combine this collateral-based sensory stimulation treatment with another therapeutic, such as tPA or recanalization. To conclude, hypertension-induced cerebrovascular impairments prevent ischemic stroke protection via the collateral-based treatment described by our lab in a rat model of essential hypertension. Further work is necessary to determine whether this treatment may be useful for patients with hypertension, and in what capacity it might prevent ischemic damage through either the enhancement of blood flow to, and rescue of, penumbral regions, or by improving delivery of tPA or other potential therapeutics to the ischemic region.

## Supporting information

S1 Fig Example of TTC staining of a treated Wistar-Kyoto rat showing no clear sign of damage. A: (top) left side view of the entire brain; (bottom) right side view of the entire brain. B: 2-mm slices of the brain in A.
(TIF)
