# Supplementary figures and images for: Hypertension prevents a sensory stimulation-based collateral therapeutic from protecting the cortex from impending ischemic stroke damage in a spontaneously hypersensitive rat model
Source: PLoS One. 2018 Oct 23;13(10):e0206291. doi: 10.1371/journal.pone.0206291 (PMC6198990; doi:10.1371/journal.pone.0206291)

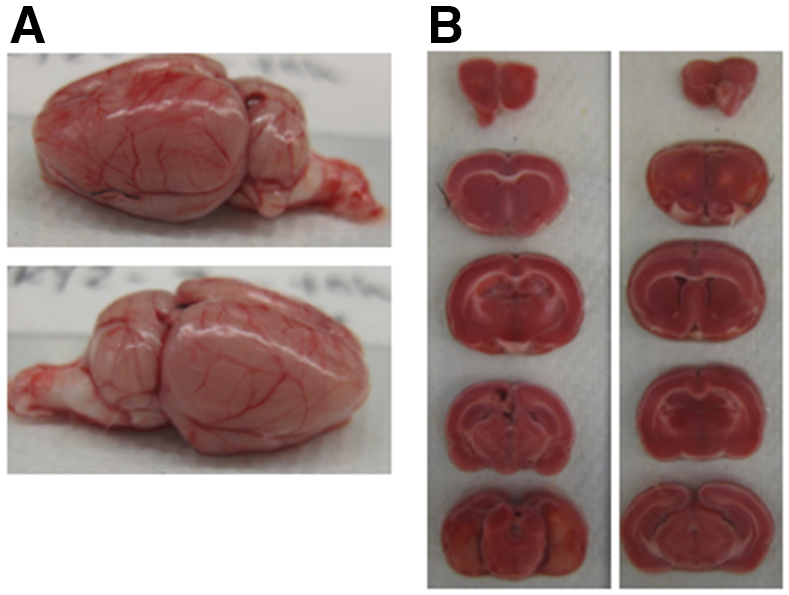

Supplement: S1 Fig — A: (top) left side view of the entire brain; (bottom) right side view of the entire brain. B: 2-mm slices of the brain in A. (TIF) [file pone.0206291.s001.tif]
